# Neural Adaptation and Behavioral Measures of Temporal Processing and Speech Perception in Cochlear Implant Recipients

**DOI:** 10.1371/journal.pone.0084631

**Published:** 2013-12-26

**Authors:** Fawen Zhang, Chelsea Benson, Dora Murphy, Melissa Boian, Michael Scott, Robert Keith, Jing Xiang, Paul Abbas

**Affiliations:** 1 Department of Communication Sciences and Disorders, University of Cincinnati, Cincinnati, Ohio, United States of America; 2 Department of Audiology, Cincinnati Children’s Hospital Medical Center, Cincinnati, Ohio, United States of America; 3 Neurology Division, Cincinnati Children’s Hospital Medical Center, Cincinnati, Ohio, United States of America; 4 Department of Communication Sciences and Disorders, University of Iowa, Iowa City, Iowa, United States of America; University of Salamanca- Institute for Neuroscience of Castille and Leon and Medical School, Spain

## Abstract

The objective was to determine if one of the neural temporal features, neural adaptation, can account for the across-subject variability in behavioral measures of temporal processing and speech perception performance in cochlear implant (CI) recipients. Neural adaptation is the phenomenon in which neural responses are the strongest at the beginning of the stimulus and decline following stimulus repetition (e.g., stimulus trains). It is unclear how this temporal property of neural responses relates to psychophysical measures of temporal processing (e.g., gap detection) or speech perception. The adaptation of the electrical compound action potential (ECAP) was obtained using 1000 pulses per second (pps) biphasic pulse trains presented directly to the electrode. The adaptation of the late auditory evoked potential (LAEP) was obtained using a sequence of 1-kHz tone bursts presented acoustically, through the cochlear implant. Behavioral temporal processing was measured using the Random Gap Detection Test at the most comfortable listening level. Consonant nucleus consonant (CNC) word and AzBio sentences were also tested. The results showed that both ECAP and LAEP display adaptive patterns, with a substantial across-subject variability in the amount of adaptation. No correlations between the amount of neural adaptation and gap detection thresholds (GDTs) or speech perception scores were found. The correlations between the degree of neural adaptation and demographic factors showed that CI users having more LAEP adaptation were likely to be those implanted at a younger age than CI users with less LAEP adaptation. The results suggested that neural adaptation, at least this feature alone, cannot account for the across-subject variability in temporal processing ability in the CI users. However, the finding that the LAEP adaptive pattern was less prominent in the CI group compared to the normal hearing group may suggest the important role of normal adaptation pattern at the cortical level in speech perception.

## Introduction

For post-lingually profoundly deafened individuals, an overall improvement in speech understanding, especially in favorable listening conditions, has been reported to be a remarkable achievement after cochlear implantation. However, the benefits of implantation vary widely across cochlear implant (CI) patients [1-3]. Furthermore, speech understanding in noise and music perception is typically poor or mediocre in most CI users [4,5]. 

Psychophysical studies have shown that the large variability in speech performance is significantly related to the individual differences in temporal processing abilities assessed with psychoacoustic tests [5-10]. By examining how the temporal properties of neural responses relate to behaviorally measured temporal processing, which has not been explored in CI users, we may better understand why some patients receive greater benefits than others from implantation. This knowledge can help to optimize the speech coding strategy and improve post-implantation rehabilitation by reproducing natural neural adaptation/recovery features. Also the neurophysiological measure can be used to objectively assess temporal processing abilities in difficult-to-test subjects if there is a significant neural-behavioral relationship.

The temporal properties of neural responses, ie., the decline of the neural response following stimulus repetition (e.g., pulse trains) and the neural recovery from preceding stimuli provide information regarding neural encoding of temporal cues [11,12]. When stimuli are repeatedly presented, neural responses decrease over time. Several mechanisms related to neural refractoriness and adaptation may contribute to this decreased response. Neural refractoriness mainly refers to the phenomenon wherein a neuron can only respond to a stimulus normally after a certain period of recovery from the preceding stimulation. Cumulative effects of sustained stimulation may result in adaptation due to depletion of neurotransmitters in synapses, either between hair cells and auditory afferents or between neurons in the auditory pathway [13-20]. Normal neural adaptation has been regarded as beneficial for sound perception because it removes redundant information and sensitizes the neurons for detecting novel stimuli [21-23]. Although both neural adaptation and refractoriness contribute to the amplitude reduction following stimulus repetition [24], it is not possible to determine the degree of contribution for each component in gross auditory evoked potentials (AEPs). Therefore, this paper will use the term “adaptive pattern” or “adaptation” as a general term to describe the neural response reduction following stimulus repetition in gross AEPs.

Most prior relevant studies examining the adaptive pattern of auditory evoked potentials in electrical hearing focused on the electrically evoked compound action potential (ECAP) [17,25, 26]. The ECAP adaptive pattern has been reported in both animals and humans [11,24]. ECAP adaptation provides the evidence to argue that the source of adaptation may include neural mechanisms in addition to inner-hair cell-auditory nerve synapse properties [14,20,25, 26], since the inner hair cell-auditory nerve synapse is bypassed in ECAP recording from CI users. 

Our group [27] was the first to examine the adaptive pattern of the late latency auditory evoked potential (LAEP) in CI users. The LAEP, which occurs at approximately 50-250 ms after stimulus onset, reflects neural activity mainly from the auditory cortex and association areas, although other brain regions may contribute when the inter-stimulus interval is long [28-30]. We found that the LAEP adaptive pattern was less prominent in CI users than in normal hearing listeners, who showed an approximately 50-60% of amplitude adaptation when the inter-stimulus interval is around 1 sec [31-32]. Moreover, we found that the amount of adaptation (adaptation index) was significantly correlated to speech perception score [27]. It is still unclear if the neural adaptation reflected by the ECAP and LAEP is related to temporal processing abilities reflected by behavioral tests (eg., gap detection test) or if the neural adaptation of both gross potentials is related to speech perception in the same group of subjects. The answer to this question has implications in improving speech coding strategy and aural rehabilitation after implantation. Previous studies have shown that the temporal properties of AEPs may be altered by manipulating the stimulus parameters or acoustic features of stimuli in NH humans and animals [19, 26]. If we are able to find clinically useful AEP tools with which to predict speech performance and temporal processing abilities, we may be able to use these tools to guide the reproduction of natural temporal features, either by manipulating the signal processing or by training patients with auditory listening tasks specific to temporal processing.

Gap detection threshold (GDT) is a commonly used measure of temporal processing resolution. Normal GDT (typically less than 20 ms) is critical for speech encoding since speech perception requires separating various speech elements that occur at a fast speed. The GDT is determined as the shortest intervals (gaps) between a pair of stimuli that a subject can perceive. Abnormal GDT is typically related to difficulties in speech discrimination and may lead to various language related abilities. In CI users, GDTs can be measured with acoustic stimuli containing gaps processed by the speech processor or with electric pulses containing gaps directly presented to the CI electrode. There are mixed results in the correlation between the GDT and speech perception in CI users. Busby and Clark [9] found a significant negative correlation between the GDT and word scores for open-set Bamford-Kowal-Bench (BKB) sentences in the auditory-visual integration condition. Muchnik et al. [8] reported that there is a trend of CI users who had open speech recognition ability tended to show lower GDTs compared to those without. Nevertheless, some researchers did not find a relation between speech performance and GDTs [33]. There has been no study examining if the GDT is related to neural adaptation in CI users.

This study focuses on neural adaptation of the ECAP and LAEP in a group of cochlear implant recipients, and compares these objective results to behavioral results of temporal processing and speech perception performance within the same group of subjects.

## Materials and Methods

### Ethics Statement

This study was approved by the Institutional Review Board of the University of Cincinnati. Participants gave written informed consent before participating in the study and were paid for their participation in this study.

### Participants

Fourteen postlingually deafened adult CI users (4 males and 10 females; age range: 24-83 years) who have used a CI in the test ear for at least 1 year were recruited through the Department of Otolaryngology at the University of Cincinnati. Among these subjects, 5 were bilaterally implanted and were tested with each ear separately. Because ECAP needed to be measured using the Neural Research Telemetry (NRT) provided by Cochlear corporation, only CI users wearing Cochlear Corporation implants (ie., Freedom and Nucleus 5) were recruited. All subjects used the Advanced Combination Encoder (ACE) strategy except one who used Spectral Peak (SPEAK) strategy. All subjects were native speakers of American English. All subjects were right-handed and had no neurological or psychological diseases except for one, who took Adderall for attention deficit hyperactivity disorder (ADHD). Clinical information of these patients is shown in Table 1. 

**Table 1:** 

**Participant**	**ECAP**	**LAEP**	**Gender**	**Ear**	**Etiology**	**Age at Test (yrs)**	**Age at Implantation (yrs)**	**Length of Auditory Deprivation (yrs)**	**Length of Implant Use (yrs)**
Sci01	x	x	F	R	Menieres	75	70	40	5
Sci08	x	DNT	F	R	Unknown	43	42	27	3
Sci16	x	DNT	F	L	Genetic	61	56	16	5
	x	DNT		R	Genetic	61	54	14	7
Sci19	x	x	F	L	Fistulas	24	17	17	3
	DNT	x		R	Fistulas	24	11	11	9
Sci20	x	DNT	F	L	Mondini Malformation	42	40	40	3
Sci21	x	x	M	L	Unknown	83	79	3	4
	x	x		R	Unknown	83	79	49	4
Sci30	x	x	M	L	Noise	65	63	18	3
Sci31	x	x	F	L	MMR	44	43	39	2
	x	x		R	MMR	44	43	39	1
Sci32	x	x	F	L	Maternal Rubella	47	43	43	5
Sci33	x	x	F	R	Unknown	75	74	64	1
Sci34	x	x	M	L	Alports Syndrome	58	45	25	13
Sci35	x	x	F	L	Unknown	59	58	55	1
Sci36	x	x	M	L	Unknown	67	66	51	1
	x	x		R	Unknown	67	66	51	1
Sci37	x	x	F	R	Unknown	37	37	36	1

Note: MMR = Measles Mumps and Rubella, DNT = Did Not Test, x = Indicates that the test was completed.

Ten healthy normal hearing (NH) subjects (1 male and 9 females; age range: 20-30 years) also participated in the study to serve as a control for LAEP data. NH subjects had audiometric hearing thresholds < 20 dB HL at octave test frequencies from 250 to 8000 Hz, normal type A tympanograms, and normal acoustic reflex thresholds at 0.5, 1, 2, and 4 kHz. 

### Procedures

CI users were tested with their clinical settings. If the participant was a bilaterally implanted patient, the data were collected from each ear separately, with the implant at the non-test ear turned off. Since all CI subjects were tested monaurally, NH subjects were also stimulated monaurally to obtain control LAEP data and behavioral data. Moreover, there is an ear difference in the temporal processing ability assessed with gap detection threshold test. Sininger & de Bode [34] reported that there was a significant left ear advantage for gap detection threshold using tonal markers in NH listeners, and the ear difference was opposite when using noise markers. Therefore, the left ear was selected as the test ear in NH listeners. During testing, the non-test ear was occluded with an E-A-R disposable ear plug (Oaktree Products, Inc.). Stimuli were presented to the test ear through a loudspeaker placed at ear level, 50 cm from the test ear at the most comfortable listening level.

### Behavioral tests

#### Random Gap Detection Test (RGDT) and RGDT-Expanded Test

The RGDT [35] is a clinically available test that assesses the auditory gap detection threshold by having the subject identify if one or two signals are heard when signal pairs are separated in time from 0 to 40 ms (0, 2, 5, 10, 15, 20, 25, 30 or 40 ms). The subtest for tonal stimuli includes tones at four frequencies (500, 1000, 2000, and 4000 Hz). Each stimulus in the signal pairs had the same frequency and the same duration (17 ms, including a 1-ms rise-fall time). The order of gap intervals was randomized. Stimulus pairs were separated by a 4.5-second interval to give subjects time to respond. The subject was required to raise 1 or 2 fingers to indicate whether he/she heard 1 or 2 tones. A practice session was presented with tone pairs at 1000 Hz followed by testing with variable gap durations. The lowest gap was detected for each of the frequencies 500–4000 Hz. When a subject failed the RGDT (no threshold was identified using RGDT), the RGDT-Expanded Test was administered in the same manner as the RGDT. The RGDT-Expanded Test is the same as the RGDT except that time intervals include 50, 60, 70, 80, 90, 100, 150, 200, or 300 ms. The stimuli were presented in the sound field through an audiometer using a CD player. 

#### Consonant Nucleus Consonant (CNC) Test

One list of CNC words were presented in quiet. The subjects were required to repeat the words they heard. The CNC test assesses the perception of isolated monosyllabic words without the syntactic and semantic cues provided in sentence-based tests. This test was used because previous studies have reported a significant correlation between temporal processing ability (gap detection or modulation detection threshold) and phoneme/word recognition [6, 36]. 

#### AzBio

AzBio [37] is a set of sentence lists that has been used to evaluate the speech perception abilities of hearing-impaired listeners and CI users. One list of 20 sentences from the multiple-talker AzBio sentence corpus was presented in quiet. These sentences range in length from 4 to 12 words and the speakers spoke in a casual style. A total of 4 speakers (2 males and 2 females) were presented for each 20-sentence list.

### Electrophysiological tests

#### ECAP measurement

The ECAP was recorded on Electrode 16 within the cochlea using the Neural Response Telemetry (NRT) incorporated into Custom Sound EP software. Although there may be differences in the adaptation across electrodes [38], we primarily selected Electrode 16 for stimulation, because this electrode typically is assigned to a frequency range involving 1000 Hz, which is close to the pulse rate preferred by most CI users [39] and also the same frequency of tone bursts used for LAEP recordings (see LAEP measurement below). If measurements from Electrode 16 were not possible due to electrode dysfunction, the recordings were made from an electrode close to Electrode 16. ECAPs were measured in response to pulses that consisted of cathodic leading, charge-balanced, biphasic current pulses and were presented in monopolar mode. Pulse width was 25 µs/phase and the interphase gap was 7 µs. 

Before recording these potentials, the dynamic range (the range of the intensity unit of electrical pulse between threshold and upper limit of comfortable loudness) at the recording electrode was checked in each subject based on their clinical mapping. Then 1000 pulse per second (pps) pulse trains were presented at different intensity levels around the most comfortable listening level and the subject was asked to estimate the loudness of the stimuli. An intensity corresponding to loudness level 7 on a 0-10-point (inaudible to too loud) numerical scale, which has been proven to be the most comfortable listening level [40,41], was used for ECAP recordings. 

**Figure 1:**
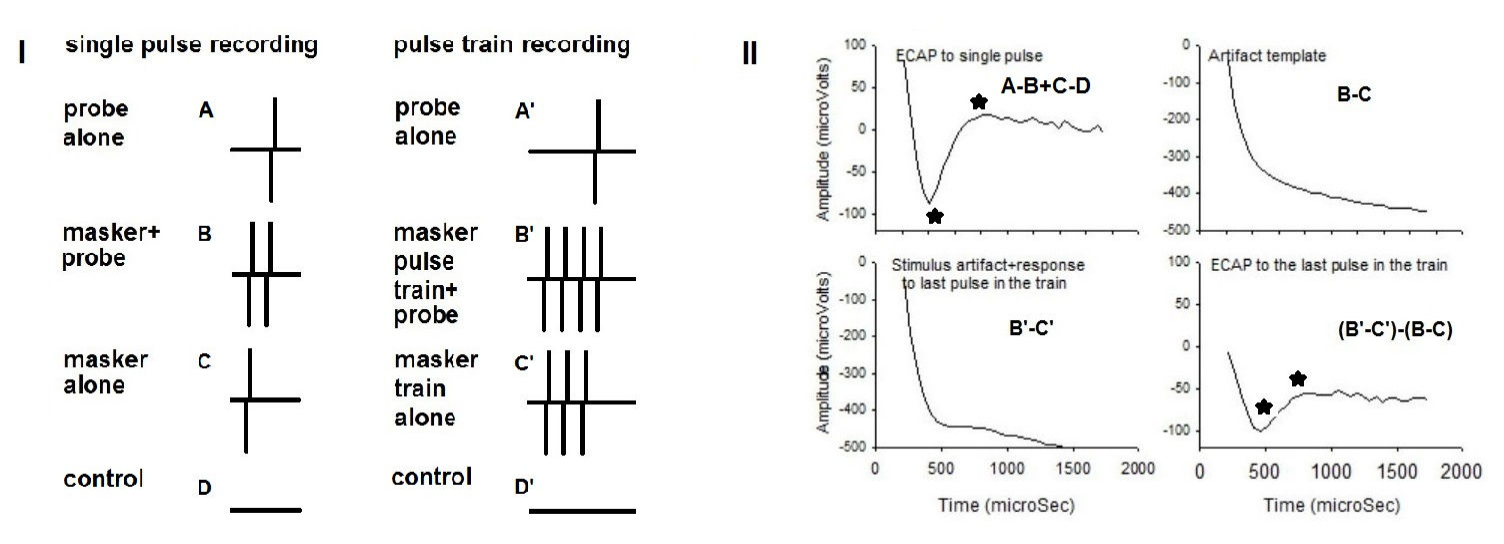
The recording conditions to obtain an ECAP in single pulse recording and in pulse train recording (I) and how the ECAP to an individual pulse in the train was derived (II). See the text in Methods section for detailed information on how to derive the ECAP to an individual pulse in the train.

This project examined ECAP adaptation evoked by a 50-ms pulse train presented at a rate of 1000 pps. Although NRT incorporates an online subtraction procedure that takes advantage of neural refractoriness to remove stimulus artifact for single-pulse ECAP [25,42], it does not allow for measuring ECAP to individual pulses in the pulse train. A previously reported approach [43] was used for this purpose. Figure 1 illustrates the procedure. Specifically, in addition to single pulse recording, the recordings were made when presenting pulse trains with different durations. The NRT uses a forward masking method that consists of recordings under 4 stimulus frames to remove stimulus artifact: probe alone (*A*), masker-plus-probe (*B*), masker alone (*C*), and zero-amplitude pulse (*D*). From the single pulse recording, the ECAP response derived by using subtraction (*A-B+C-D*) was used as the response to the 1^st^ pulse in the train when evaluating adaptation. Also the single pulse recording provides an artifact template that was derived by subtracting the recording frame for the masker alone condition from the recording frame for the masker-plus-probe condition (B-C). In the pulse train recording, the last pulse in the train was treated as the probe and the preceding pulses were treated as the masker pulse train. After the data were collected, the recording from the masker pulse train-alone condition was subtracted from the masker pulse train-plus probe condition (*B’-C*’) to derive an ECAP to the last pulse in the train along with the stimulus artifact (*B’-C*’). Then the artifact template from single pulse recording was subtracted from to obtain the ECAP to the last pulse in the train [*(B*’*-C*’)*-(B-C*)]. By changing the duration of the pulse train (thereby the number of pulses), we were able to obtain ECAP evoked by individual pulses in the train (Figure 1). 

Time constraints prohibited the recording of an ECAP response to each of the pulses in the masker pulse trains. Therefore, 13 different masker pulse-train lengths were chosen to assess the degree of auditory-nerve adaptation. These pulse trains contained 2, 3, 4, 5, 6, 23, 24, 25, 26, 48, 49, 50, and 51 pulses, respectively. In addition, single-pulse ECAP recording was measured 5 times (the first and the last 4 recordings). The average of the 5 recordings was used to provide the ECAP to the first pulse in the train for adaptation measure and the artifact template. Therefore, there were a total of 18 recordings for each subject.

#### LAEP measurement

The stimuli were 1 kHz tone bursts (60 ms, 10 ms rise/fall time) generated and presented using STIM^2^ software through the Neuroscan workstation (Compumedics Neuroscan, Inc., Charlotte, NC). The stimulus-train paradigm used in this study was similar to that in previous studies [31, 32, 44, 45]. The tone bursts were presented in 30 trains consisting of 5 tone-bursts each, with inter-stimulus intervals (ISIs) of 0.7 sec and inter-train intervals (ITIs) of 15s. The stimuli were presented at an intensity level corresponding to a loudness level of approximately 7. At least 6 recordings (180 trials for each stimulus in the train) were collected from each subject.

The electroencephalographic (EEG) recordings were performed using a 40-channel system (NuAmps, Compumedics Neuroscan, Inc., Charlotte, NC). Electrode placement was based on the International 10-20 system, using an Electro-Cap placed on the head of the participant, with the contralateral earlobe as the reference. This procedure has been found to reduce the stimulus artifact in some CI users (McNeill et al., 2007). Electroocular activity (EOG) was monitored so that eye movement artifacts could be identified and rejected during the offline analysis. Approximately 1-3 electrodes located directly over or closely surrounding the implant transmission coil were not used. Electrode impedances in the remaining electrodes were kept at or less than 5 kΩ. Continuous EEG recordings were collected from participants using the SCAN software (version 4.3, Compumedics Neuroscan, Inc., Charlotte, NC) with a band-pass filter setting from 0.1 to 100 Hz, and an analog-to-digital-converter (ADC) sampling rate of 1000 Hz. 

### Data Analysis

#### Behavioral tests

The GDTs (ms) at 4 different frequencies (0.5, 1, 2, and 4 kHz) were obtained. The composite GDT (CGDT) value was also calculated by averaging the values for the tested frequencies. CNC words and AzBio sentences were evaluated using the percent correct score. The average score of CNC and AzBio outcomes was used for each subject to represent the speech perception performance.

#### ECAP

After the ECAP data collection, the data were exported and entered to Sigmaplot software program to do off-line analysis. Further analysis was performed to derive the ECAP for each pulse in the train using the aforementioned pulse-train subtraction technique. The averaged ECAP of the 5 single pulse recordings was used as the response to the 1^st^ pulse in the train. The negative and positive peaks were selected for each ECAP as illustrated in Figure 1. The amplitude of the ECAP was measured between the negative and positive peaks. The ECAP was compared between the single pulse (1^st^) and later stimuli in the train. 

#### LAEP

EEG data from both CI and NH subjects were processed using SCAN software (Compumedics Neuroscan, Inc., Charlotte, NC). The data were epoched with a 100-ms pre-stimulus time and a 500 ms post-stimulus time, the repeated recordings were combined, and baseline adjustment was performed based on data in the pre-stimulus time of each stimulus in the train. Data was visually checked and the epoches that show excessive noise such as the responses from the muscle artifact were removed.

In order to minimize stimulus artifacts, the epoched data were concatenated and imported to the EEGLAB Toolbox (EEGLAB, San Diego, CA), an online open source toolbox (freely available from http://sccn.ucsd.edu/eeglab), to minimize the stimulus artifact using Independent Component Analysis [46, 47]. The ICA model decomposes the EEG dataset into mutually independent components, including those from artifactual and neurophysiologic sources. Then an iterative process changes the weights and directions of the vectors in a mixing matrix until maximum independence is identified from the higher order statistics. The stimulus artifact components were identified using topographic 2-D maps with the criteria of CI artifact described in a previous study [47]. Simply, the main feature of the artifact components is that the artifact starts slightly after the stimulus onset and ends slightly after the stimulus offset, with a square shape during the stimulus presentation. Another feature of artifact components is that scalp projections of the activity display a centroid on the side of the implanted device. After identifying stimulus artifact components, these components were linearly subtracted from the epoched EEG data. The remaining components were then constructed to form the final epoched EEG recording, which was later filtered (0.1-30 Hz, 12 dB/octave). Finally, averaged responses were derived independently for each tone burst within the train across the total number of train presentations. For each LAEP, the N1 and P2 peaks were labeled and the N1-P2 peak-to-peak amplitude was used for the amplitude of the LAEP. In CI users, the N1 was defined as the most negative peak around 100 ms after stimulus onset and the P2 was the positive peak following the N1. In CI users, the N1 may occur later. Therefore, we looked for the typical morphology of N1-P2 complex before identifying the N1. Because the LAEP amplitude is the largest for electrode Cz and becomes progressively smaller for electrodes distant from Cz, we restricted the later analysis to measures from Cz.

## Results

Among the 14 CI users, the numbers of subjects who finished behavioral tests, ECAP recording, and LAEP recording were 14 (19 ears), 14 (18 ears), and 11(15 ears), respectively (See Table 1 for details). The average GDTs for the 4 tested frequencies (0.5, 1, 2, and 4 kHz) were 24.71 ms (SD=17.72), 24.53 ms (SD=18.11), 23.24 ms (SD=16.10), and 27.35 ms (SD=22.51), respectively. The average composite GDT was 24.96 ms (SD=16.21). The average score was 62.40% (SD=23.41) for CNC and 80% (SD = 26.27) for AzBio. Note that the average was based on all the ears included because all tests were performed monaurally even in the subjects who were bilaterally implanted. In NH listeners, the average GDTs for the 4 tested frequencies were 5.50 ms (SD=4.62), 5.10 ms (SD=2.92), 5.80 ms (SD=4.47), and 5.90 ms (SD=3.87), respectively. The average composite GDT was 5.58 ms (SD=3.23). Because the composite GDT data were not normally distributed, Mann-Whitney Rank Sum Test was performed. The difference between the two groups was significant (T = 55, p<0.05).

**Figure 2:**
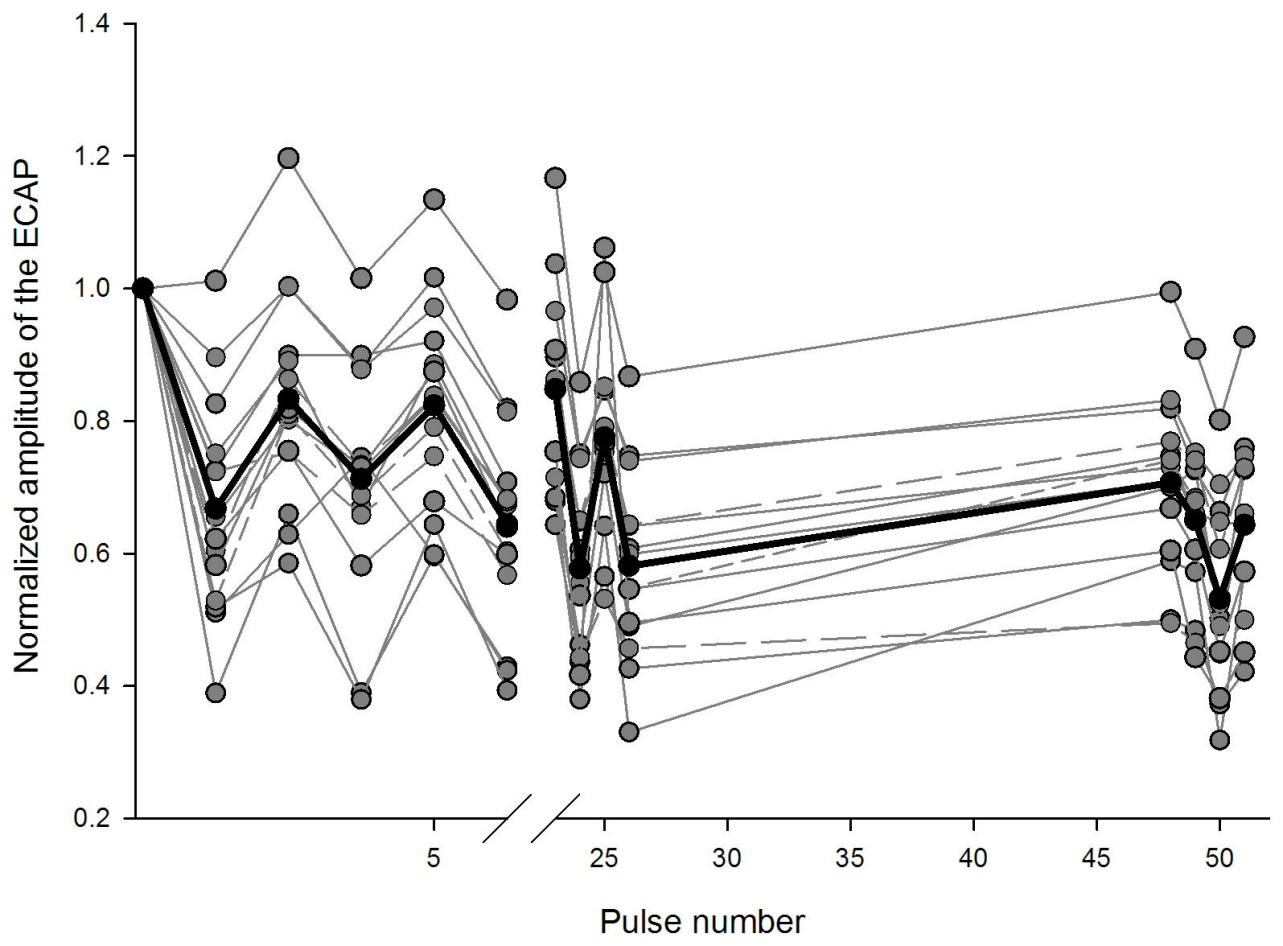
Normalized ECAP amplitudes as the function of pulse number in the 50-ms pulse train. Each trace in dark gray represents data from one subject and the trace in black represents the mean data. Normalized ECAP amplitude was calculated by dividing the ECAP amplitude to pulse 2 to pulse 51 in the train by the ECAP amplitude in the single pulse recording. An alternating pattern is observed in every subject.

ECAPs were successfully revealed after artifact removal in all ears except 4. Figure 2 shows the normalized ECAP amplitude as a function of the pulse number in the train. The normalized amplitude was derived by dividing the ECAP amplitude evoked by individual pulses to the amplitude evoked by the first pulse in the train. The alternating pattern, in which the amplitude for odd numbers is larger than that for even numbers of pulses, existed for all subjects, despite the different degrees of alternation. Note that the ECAP in all subjects except one displayed amplitude adaptation. In order to quantify the adaptation amount, the adaptation index (AI_ECAP_) was calculated by using the equation as follows: AI_ECAP_=1-A_last3_/A_1_, in which “A_last3_” is the average amplitude of ECAPs for the last 3 pulses. 

**Figure 3:**
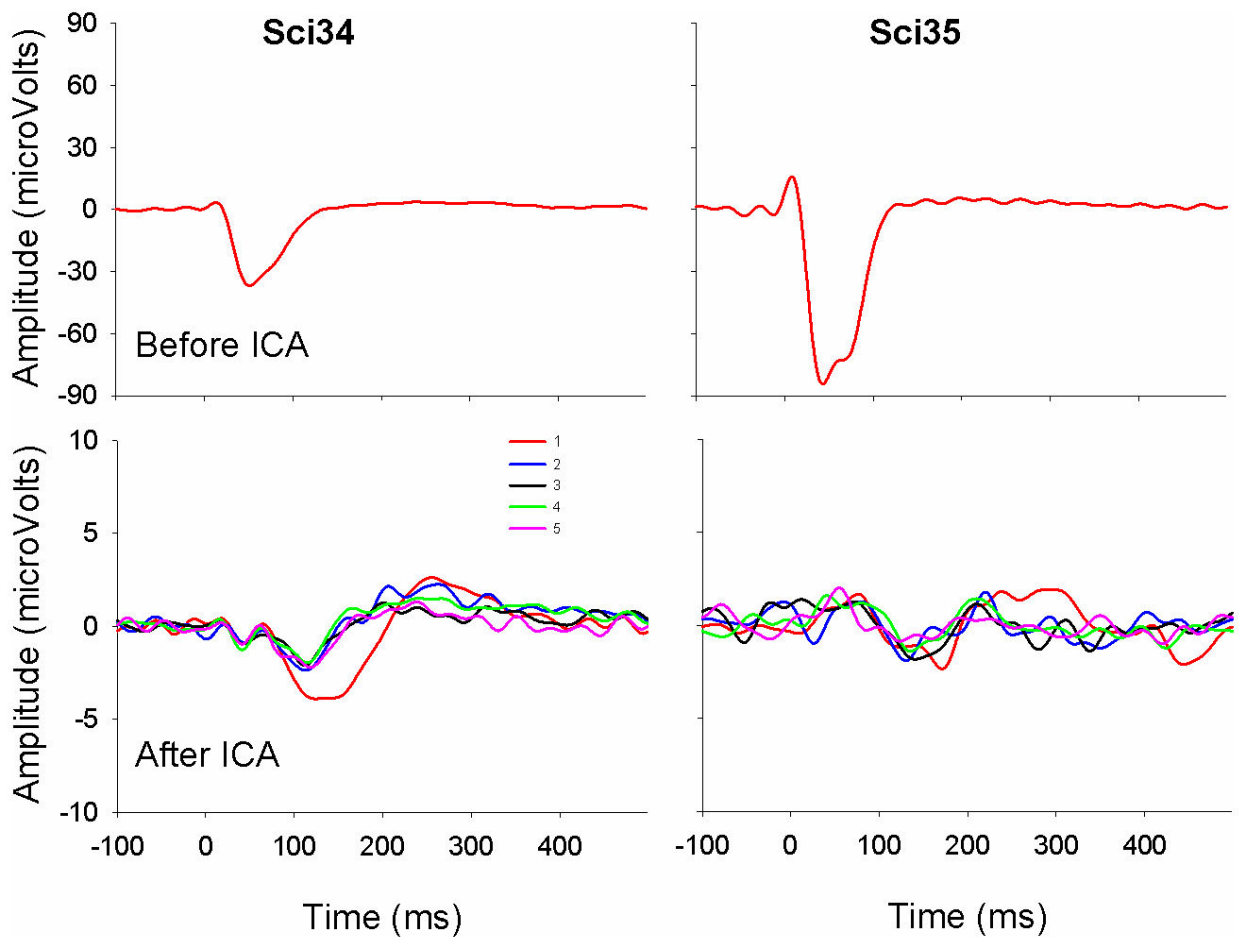
Examples of the EEG responses contaminated by stimulus artifacts (top plots) and the LAEPs after artifact removal (bottom plots) at the vertex electrode recorded from two CI subjects. It can be seen that the CI subject on the left shows a more prominent adaptive pattern than the CI subject on the right (the response to the 1^st^ stimulus is larger than the responses to the later stimuli in the train). Note that the y-axis of the top plots before ICA procedure has a different scale compared to other plots due to the large artifacts.

Figure 3 shows an example of LAEPs evoked by tone burst trains in two CI users after artifact removal using ICA. Note that the y-scales of the top and bottom plots were different due to the large artifacts in the former. It can be seen that the artifact was successfully minimized in the CI users and LAEPs evoked by individual stimuli in the train were revealed. The CI user on the left displays an adaptive pattern similar to that in the NH listeners to be presented later, with the largest amplitude for the first stimulus and similarly smaller amplitude for the rest of the stimuli in the train. By contrast, the CI user on the right does not have an obvious adaptive pattern.

**Figure 4:**
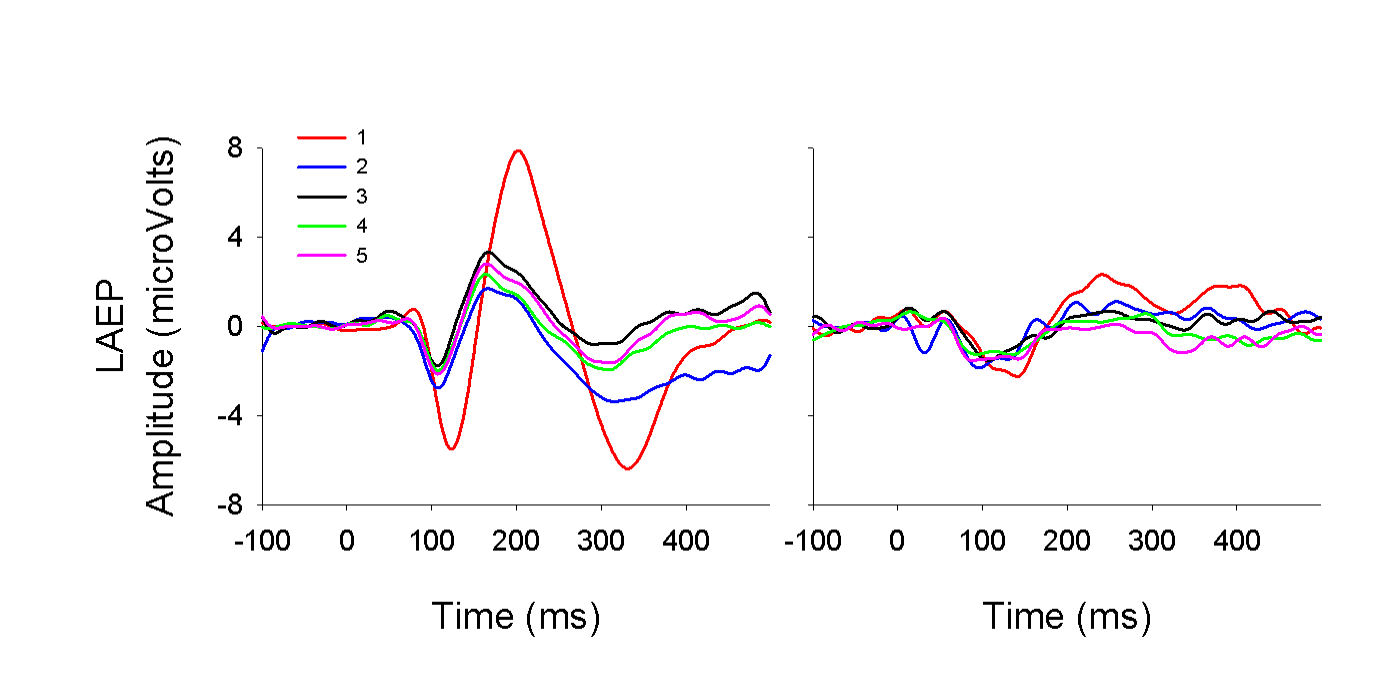
The averaged LAEP waveforms evoked by individual stimulus in the train for the NH group (left) and the CI group (right).

Figure 4 shows the averaged LAEPs evoked by individual stimuli in the train in the CI group and the NH group. In NH group, the adaptive pattern of the LAEP is prominent, with an approximately 60% of amplitude reduction. In CI group, the adaptive pattern is less prominent, with an amount of approximately 40% of reduction. The most prominent features in CI group relative to NH group are: 1) the response to the first stimulus in the train is much smaller, and 2) there is less adaptation in the LAEP in CI users. The adaptation index (AI_LAEP_) was calculated to quantify the amount of adaptation using the equation as follows: AI_LAEP_=1-A_last3_/A_1_, in which “A_last3_” is the average amplitude of LAEPs for the last 3 stimuli in the train. 

**Figure 5:**
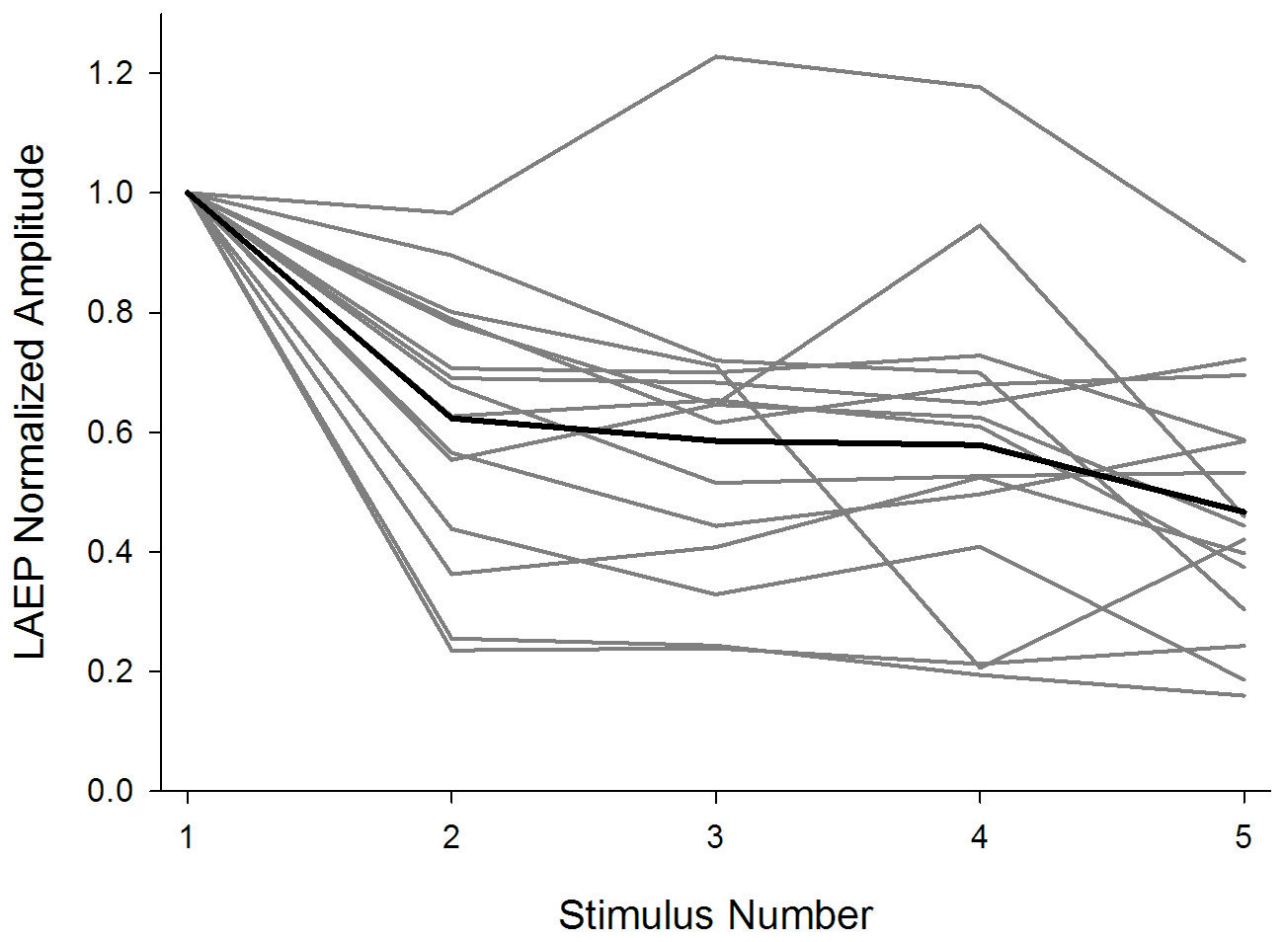
The normalized amplitude of the LAEP as a function of stimulus order for individual CI subjects. Each trace in dark gray represents data from one subject and the trace in black represents the mean data. Normalized LAEP amplitude was calculated by dividing the LAEP amplitude to an individual stimulus in the train by the LAEP amplitude evoked by the first stimulus in the train.

Figure 5 displays CI subjects’ normalized N1-P2 amplitude as a function of stimulus order. Each gray trace shows data collected from one subject. The mean data are also shown in the black trace. Except for one subject displaying enhanced amplitude for later stimuli in the train compared to the first stimulus, all subjects displayed an adaptive pattern in the LAEP. Note that the subject displaying the enhancement in the LAEP is not the same as that displaying enhancement in the ECAP.

**Figure 6:**
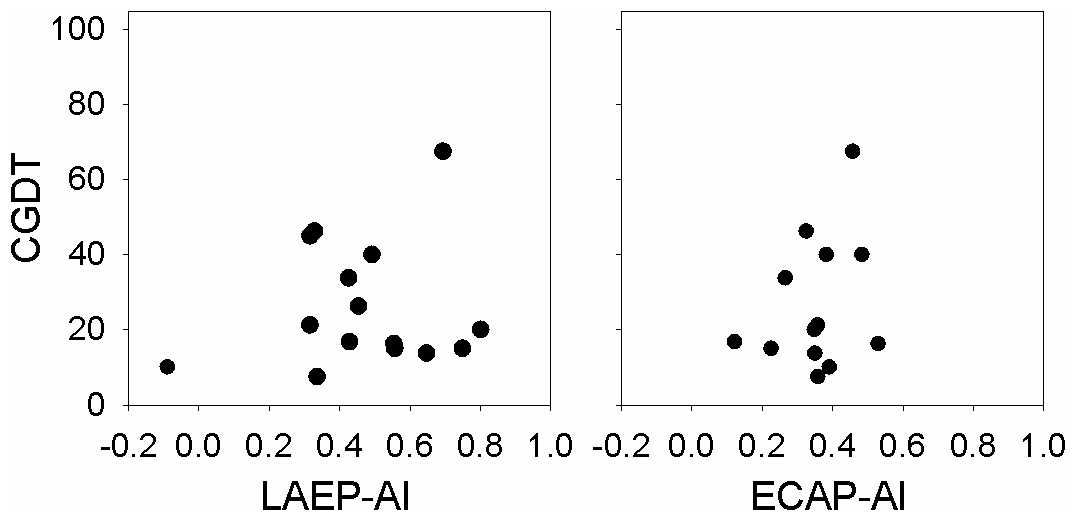
The correlation between adaptation index and the CGDT in CI users.

**Figure 7:**
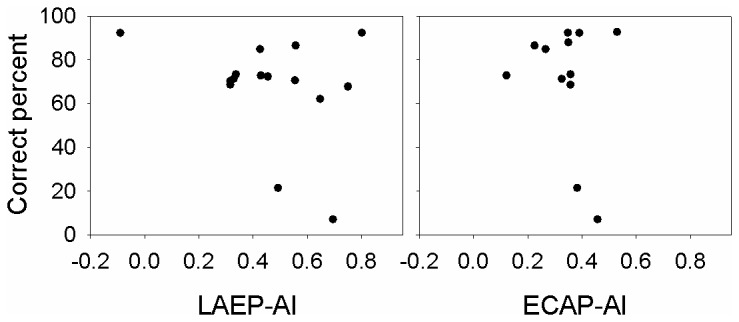
The correlation between adaptation index and speech perception scores (the average scores of CNC and AzBio) in CI users.

The correlation between the CGDT and the adaptation index of the ECAP and LAEP (Figure 6) was not significant (p>0.05). Figure 6 shows the scatter plots of the CGDT and the adaptation index of the ECAP and LAEP, respectively, as a function of AI. There was no significant correlation between the adaptation index and the average score of CNC and AzBio (p>0.05). Figure 7 shows the scatter plots of the CGDT and speech perception scores (the average scores of CNC and AzBio), respectively, as a function of AI. 

**Figure 8:**
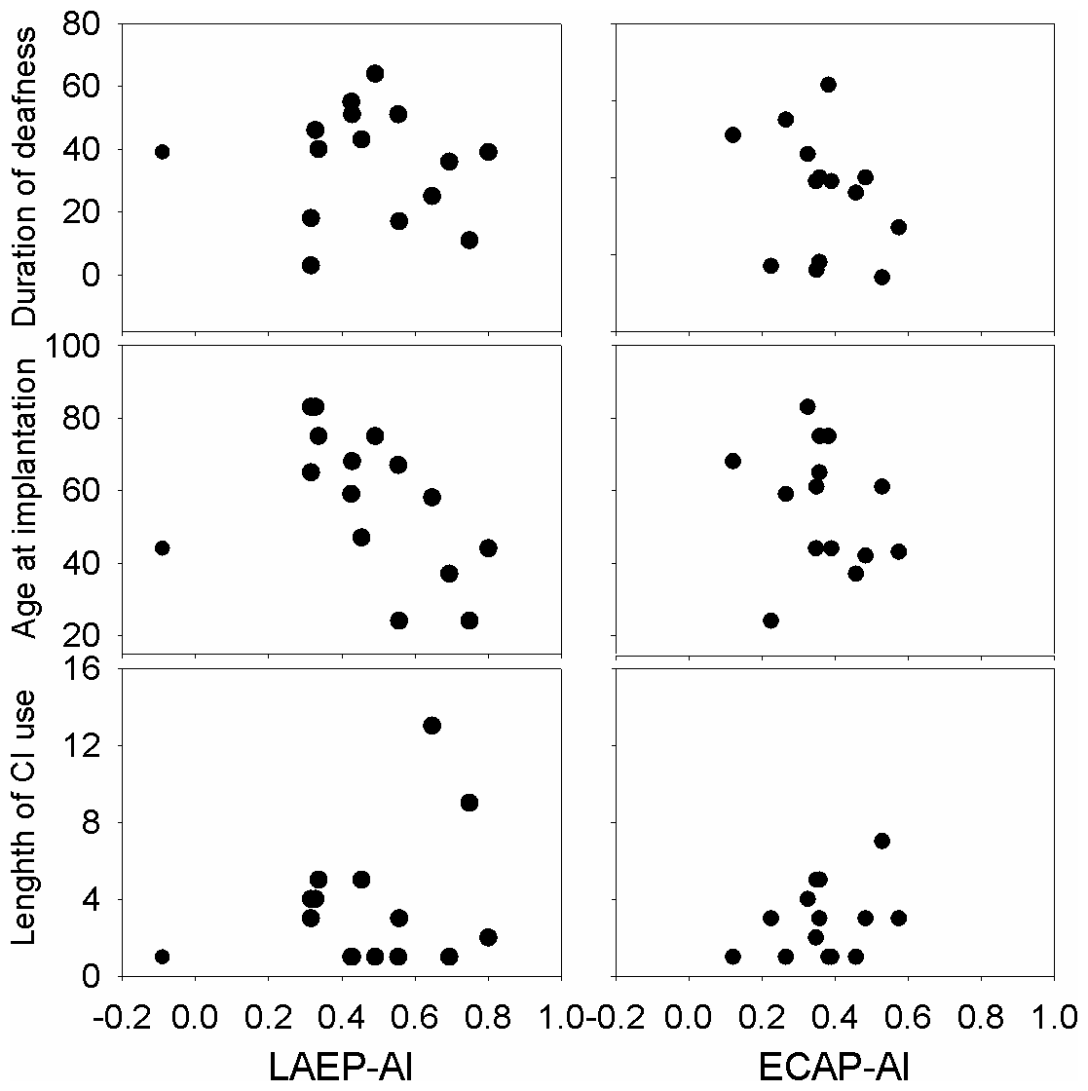
The correlation between adaptation index and demographic factors including the duration of deafness, the age at implantation, and the length of CI use. Among all the scatter plots, the correlation between AI_LAEP_ and the age of implantation is statistically significant, if one outlier that shows amplitude enhancement rather than adaptation is not included in the analysis.

Figure 8 shows the correlation between some demographic factors and the adaptation index of the ECAP and LAEP. The demographic factors included the duration of deafness, the age of implantation, and the length of CI use. Pearson correlation analysis showed that there is a significant negative correlation between the age at implantation and LAEP adaptation index if one outlier data point that shows an enhancement rather than adaptation in the LAEP is excluded from the analysis (R= -0.74, p<0.05).

In summary, both ECAP and LAEP display adaptive patterns in most CI users, with a substantial variability in the amount of adaptation. The adaptation index is not correlated to gap detection thresholds. There was no statistical significance between the adaptive index and speech performance. Subjects who are implanted at a younger age are likely to have a larger LAEP adaptation index.

## Discussion

The present study examined whether the temporal properties of neural responses can be used to account for the across-subject variability in temporal processing abilities and speech perception in CI users. We did not find significant correlations between the neurophysiological and behavioral measures.

### GDTs

In the literature, while most studies measured GDTs using single-electrode stimulation [33,48], only a few examined GDT using acoustic stimuli through clinical processors [8, 49]. The GDT measured via clinical processors reflects limitations imposed by both electrical stimulation and processing strategy, which is similar to what occurs for sound perception in CI users’ daily life. GDTs recorded using the above two approaches appear to be different. Specifically, with direct electrical stimuli, postlingually-deafened CI users’ GDT is similar to that in NH listeners [33, 50], with a range of 1.8 to 32.1 ms for different pulse rates [9]. If acoustic stimuli are used, the GDT in prelingually-deafened CI subjects averages 30 ms ranging from 4 to 128 ms [49]. The average of 26.28 ms for CI users with a range of 7.50 to 67.50 ms in the current study is consistent with the findings in prior studies. The difference in GDTs between the direct electrical stimulation and CI processing may indicate that the signal distortion associated CI processing can exacerbate the deficits of temporal processing. Further studies should be conducted to determine to what extent the CI processing affects temporal processing abilities of CI users.

### ECAP

The ECAP adapts in amplitude following repetition of stimulus. Prior studies reported that the adaptation occurs when stimulus rate is as low as 80 pps [38] and the amount of adaptation is greater when rate increases [11]. In the current study, ECAPs evoked by 1000 pps trains did display adaptive patterns and the adaptation index is 0.12-0.57 (see Figure 7). Hay-McCutcheon et al. [43] reported that the range of the adaptation index of the ECAP was approximately 0.1-0.5, with an outlier of 0.8 (Figure 10 of Hay-McCutcheon et al. [43]). Although the two studies used somewhat different equations for calculation (Hay-McCutcheon et al. used the average of the last 10 ECAPs but we used only last 3 ECAPs for the calculation of AI), the adaptation index in these two studies is similar because either the last 10 or 3 ECAPs can represent the response at the asymptotic level. An alternating pattern in ECAP amplitude as a function of stimulus order was obvious across subjects. Several prior studies examining ECAP adaptation have reported this phenomenon. For instance, ECAP displays alternating pattern for a rate ranging from 400 to1500 pps [24, 51] and this pattern has been regarded as the result of refractory properties of individual nerve fibers. The alternation diminished and further ceased for faster rates (>2000 pps), which reflects cross-fiber desynchronization. The rate for desynchronization/stochasticity is affected by cochlear regions, stimulus intensity and varies across subjects [24]. 

Previous studies have not successfully found the correlation between ECAP adaptation measures and perceptual performances of CI users. Hay-McCutcheon et al. [43] examined ECAP adaptation to a 1000 Hz pulse train in adult Nucleus CI recipients, and found no relation between the amount of adaptation and psychophysical measures of temporal integration, the ability to use acoustic information provided in a time window in order for sound perception (ie., decreased behavioral threshold as a result of the increase of stimulus duration). Clay & Brown [38] recorded a series of ECAPs at stimulation rates of 15, 80, and 300 pulses per second (pps). The authors reported no significant correlation between ECAP adaptation and word recognition scores. 

#### LAEP

A number of studies have reported adaptation of the LAEP in NH listeners [13, 32, 44, 52-54]. Authors typically have found that the amplitude reduction can be approximately 50% over the first several stimuli before it reaches asymptotic amplitude, which is lower for shorter ISIs than for longer ISIs [31, 55]. LAEP amplitude does not change significantly for ISIs longer than 10 s [55]. 

Our group was the first to report LAEP adaptation in CI users [56]. We reported that the LAEP adaptive pattern in CI users (mostly stimulated monaurally) was less prominent than in NH listeners (stimulated binaurally) reported in Zhang et al. [57]. The current study first reports a direct comparison between CI and NH subjects with monaural stimulation. The finding of less prominent adaptive pattern in CI users compared to NH listeners in the current study is consistent with that reported in Zhang et al. [56]. One explanation for the reduced amount of adaptation in CI users is that neural degeneration may have altered the adaptation/refractory features of neural responses [58]. This explanation is supported by a significant correlation between AI_LAEP_ and the age at implantation, which is a factor affecting the degree of neural degeneration. An alternative explanation is related to the reduced amplitude of the LAEP evoked by the 1^st^ stimulus in the train and the lack of further amplitude decrements in the LAEP evoked by later stimuli. The LAEP evoked by stimuli presented with a long ISI, ie., the 1^st^ stimulus in a train, is mainly due to the “non-specific” component generated from regions outside of the auditory cortex, while the LAEP evoked by stimuli at a fast and fixed rate is mainly generated from the auditory cortex [28]. These “non-specific” components need a longer time to recover from refractoriness compared to the component in the auditory cortex. The smaller LAEP evoked by the first stimulus in the train in CI users may indicate that the “non-specific” component of the LAEP is more compromised than the component in the auditory cortex due to long-term deafness. 

The above speculation that the reduced contribution of the non-specific generator to the LAEP leads to the less prominent adaptation in CI users compared to NH listeners can also be used to explain the less obvious latency reduction in the N1 for the 2^nd^ stimulus relative to the 1^st^ stimulus of the train (Figure 3 and 4) in CI users compared to NH listeners. Previous studies reported that N1 is generated from at least two cortical generators that are spatially and temporally distinct [44, 59, 60]. One component has a shorter latency and a shorter refractory period of 1 to 3 s, whereas the other component has a longer latency and a much longer recovery period [28]. These two N1 components can be distinguished on the basis of their distinct refractory properties [28, 61]. The component with a shorter latency can be elicited by stimuli at any rates and the component with a longer latency can be elicited by stimuli at much slower rates [60, 62]. Therefore, the reduction of N1 latency for the 2^nd^ stimulus relative to the 1^st^ stimulus in NH listeners is likely to be related to faster recovery of neurons with shorter latencies when neurons with longer latencies are still influenced by refractoriness. In contrast, CI users’ damage of long-latency generator leads to less prominent latency reduction for the 2^nd^ stimulus relative to the 1^st^ stimulus in the train.

The combination of the current finding of less adaptation in CI users relative to NH listeners and findings in prior psychophysical and EEG studies provides clearer mechanisms of electric hearing in CI users. Using forward masking paradigm, Chatterjee [63] reported that the psychophysical recovery from forward masking is faster in poorer CI performers than good performers. The recovery functions display a rapid and a slow component and temporal integration influences the slow component more than the rapid component. Their two-exponential model suggests that the shorter the masker duration, the lesser the temporal integration, the less the contribution of the slow component, and the faster the recovery from masker. Therefore, Chatterjee’s study suggested that the temporal integration of CI users is damaged. A previous electrophysiological study in CI users in our lab [56] reported that the recovery function of the LAEP in CI users appeared to be faster in general compared to NH listeners. One possible explanation of this phenomenon is that CI users may have a reduced contribution of the non-specific generator to the LAEP and thus only the fast recovery of the generator in auditory cortex is shown. Taken together, the less prominent adaptive pattern, the faster recovery of LAEP, and the faster psychophysical recovery function in CI users, suggests that the temporal integration ability, which is likely related to the slow recovering neural generators, could be compromised in this population. Much shallower temporal integration functions have been seen in CI users by other researchers [64]. Further research involving both temporal integration measures and EEG source analysis may help confirm our speculation. 

#### Neural adaptation and behavioral measures

The current study did not find a significant correlation between either ECAP adaptation or LAEP adaptation and gap detection thresholds. Several possible reasons are speculated. First, it is possible that there is an interaction of the effects of stimulus parameters on different measures. Gap-detection thresholds depend on stimulus intensity in both acoustic and electric conditions and the adaptation of both ECAP/LAEP can also be affected by stimulus intensity [57, 65, 66]. Although we presented stimuli at the most comfortable listening level for all tests, the stimulus intensity corresponding to their most comfortable listening levels may be different within the dynamic range for subjects. It is possible that the variability of ECAP/LAEP adaptation and gap detection thresholds resulting from different stimulus intensities led to the lack of correlation. The second possible reason is the low sensitivity of measures used for examining the neural-behavioral correlation. For example, the recovery function of neural responses has been reported to indicate encoding of temporal cues [67]; the behavioral measure of modulation detection threshold has been used to assess temporal processing abilities [10, 68]. It is worthwhile to use other measures of neural responses and temporal processing to reassess the neural-behavioral correlation. 

Although our group reported a significant correlation between LAEP adaptation index and speech perception performance in Zhang et al. [56], the current study did not find such correlation. This may be related to the difference in the speech materials used and the subjects recruited in the two studies. Zhang et al. [56] used the average of CNC word recognition in quiet, HINT sentence recognition in quiet, and HINT sentence recognition at 10 dB signal-to-noise ratio (SNR) for speech perception score. The current study used CNC and AzBio. The HINT sentences in quiet and in a background of fixed-level noise have been thought to be relatively easy compared to AzBio [69]. AzBio appears to be a more reasonable test for speech perception than HINT because CI users’ performance in AzBio displays a wide range of distribution while the HINT test, especially in quiet, suffers from ceiling effects. Further research needs to be conducted using other speech tests suitable for CI users to determine if the correlation between LAEP adaptation index and speech perception exists. Moreover, the current study involved more binaural CI users for LAEP test (4/11, see Table 1) compared to Zhang et al. [56] (2/10). Because the two ears of binaural CI users were tested separately in both studies, there was heavier distribution of data results from the same individuals in the current study. 

Despite the differences between the current study and Zhang et al. [56], the finding that less prominent LAEP adaptive pattern in CI users compared to NH listeners was consistent in both studies. This finding indicates the positive role of normal LAEP adaptation in speech perception. This finding supports a view in prior studies that proper neural adaptation following stimulus repetition is useful for speech perception by sensitizing the auditory system for novel stimulus detection [70]. 

We observed a rather high variability among individual CI users for ECAP, LAEP, and GDT data. Part of this variability may be related to CI users’ demographic factors such as the age at implantation, the duration of deafness, and the length of CI use etc. The correlation analysis found that there is a significant correlation between the LAEP adaptation index and the age at implantation if one outlier data point representing response enhancement rather than adaptation is not included for the analysis. This suggests that CI users who have a greater LAEP adaptation index tended to be the ones implanted at a younger age. Implantation at a younger age may prevent degeneration of auditory system and therefore results in a more normal LAEP adaptive pattern. The results indirectly indicate the positive role of LAEP adaptive pattern in CI performance.

### Implications and future studies

Our LAEP data showed that LAEP adaptive pattern in CI users is less prominent than in NH listeners. These results may contribute toward improved speech processor design and rehabilitation in the post-implantation stage. Geurts and Wouters [21] suggested that neural adaptation feature at the nerve level should be incorporated into CI speech processing, eg., by increasing the amplitude of the speech envelope at sudden intensity increases. We suggest that improving the speech processing may involve creating a normal-like LAEP adaptive pattern through electric stimulation. For post-implantation rehabilitation, we suggest that materials rich in temporal changes should be used for auditory training. Kilgard and Merzenich [71] found that the neural adaptation pattern in rat primary auditory cortex becomes more prominent after training the rats with such stimuli. Finally, neural adaptation at both peripheral level and cortical level cannot serve as an objective measure of temporal processing ability reflected by gap detection threshold. More research needs to be done to determine if there is a correlation between the LAEP adaptation and speech perception in CI users. 

### Limitations

The main limitation in this study is that the NH group aged 20-30 years while the CI group aged 24-83 years. There is ample evidence that temporal processing alters as a function of age. Kumar and AVS [72] reported that, as age increases, temporal processing abilities measured with tests including gap detection deteriorate and the rate of deterioration becomes faster after 70 year of age. Although the current study provides the basic information regarding differences between abnormal and normal patterns of LAEP adaptation and the correlation between adaptation measures and GDT/speech perception, age-matched control subjects should be used in future studies to rule out age effects. 

## Conclusions

This project investigated if the adaptation of the ECAP and the LAEP can account for the across-subject variability in behavioral performance of temporal processing and speech perception in CI recipients. The results showed both ECAP and LAEP displayed adaptive patterns, with a substantial variability across subject. No significant correlations between neural adaptation and behavioral performance were found. CI users showing more LAEP adaptation were likely to be those implanted at an early age than CI users with less LAEP adaptation. The finding that the LAEP adaption was less prominent in the CI group compared to the normal hearing group may suggest the important role of normal adaptation at the cortical level in speech perception. We proposed that, in order to improve CI outcomes in speech perception, it is necessary to create a normal-like LAEP adaptive pattern, either by manipulating the stimulus parameters in CI speech processing or using appropriate auditory training material for post-implantation rehabilitation.
